# Transfection of Antisense Oligonucleotides Mediated by Cationic Vesicles Based on Non-Ionic Surfactant and Polycations Bearing Quaternary Ammonium Moieties

**DOI:** 10.3390/ijms18061139

**Published:** 2017-05-26

**Authors:** Judith Mayr, Santiago Grijalvo, Jürgen Bachl, Ramon Pons, Ramon Eritja, David Díaz Díaz

**Affiliations:** 1 Institut für Organische Chemie, Universität Regensburg, Universitätsstr. 31, Regensburg 93053, Germany; judith.mayr@chemie.uni-regensburg.de (J.M.); bachl_j@web.de (J.B.); 2 Institute of Advanced Chemistry of Catalonia-Spanish National Research Council (IQAC-CSIC), Jordi Girona 18-26, Barcelona 08034, Spain; sgrgma@cid.csic.es (S.G.); ramon.pons@iqac.csic.es (R.P.); 3 Networking Centre in Bioengineering, Biomaterials and Nanomedicine (CIBER-BBN), Jordi Girona 18-26, Barcelona 08034, Spain

**Keywords:** antisense oligonucleotide, cationic polymers, cationic vesicles, ionenes, transfection, polycations, polyplexes, quaternary ammonium

## Abstract

Three different ionene polymers with varying quaternary ammonium moieties were used as a proof of concept for the formulation of antisense oligonucleotides, which are capable of inhibiting *Renilla* luciferase messenger ribonucleic acid (mRNA). Cationic vesicles, consisting of cationic polymer, antisense oligonucleotide (*Luc*) and non-ionic surfactant polysorbate 80, were investigated regarding their ζ potential, cytotoxicity and transfection efficiency. Deoxyribonucleic acid- (DNA) forming complexes in the presence of cationic vesicles were also investigated in terms of small-angle X-ray scattering (SAXS). The studied cationic vesicles showed very little, if any, toxicity against HeLa cells. Transfection abilities proved to vary strongly depending on the present quaternary ammonium moiety.

## 1. Introduction

The last decades have witnessed a growing interest in the use of synthetic oligonucleotides for the inhibition of gene expression [1] as an alternative to the classical small molecule drugs to treat diseases. Examples for such oligonucleotides are antisense oligonucleotides, short interfering RNAs (siRNAs) [2], aptamers [3], DNA/RNAzymes and antisense-induced exon skipping [4,5]. In order to achieve the desired inhibition some obstacles must be solved. The first problem to solve is the high sensitivity of oligonucleotides towards degradation by serum nucleases. This has been partially solved by the use of chemical modifications of the oligonucleotides at the sugar ring and/or at the phosphate backbone in order to increase their biostability [6,7]. Cellular uptake of polyanionic oligonucleotides across the negatively charged cell membrane constitutes another problem. Although it has been shown that viral vectors are highly efficient for the transfection of plasmid DNA, there are still concerns about immunogenicity or recombination of oncogenes. Several alternatives for viral transfection methods have been developed including formulations as a very fast and simple method [8].

In 1987, Felgner and co-workers reported the first transfection experiment. They used the cationic lipid *N*-[1-(2,3-dioleyloxy)propyl]-*N*,*N*,*N*-trimethylammonium (DOTMA) for carrying out an efficient DNA transfection protocol [9]. Since this, a huge variety of cationic lipids for formulation of oligonucleotides have been described [10,11]. Even though they comprise promising tools for nucleic acid delivery there are several aspects to be considered. Cationic lipids have the tendency to interact with plasma proteins, which has a detrimental effect on transfection efficiency [12]. In addition, high positive net charge of the formulation can cause toxicity. Therefore a demand for new formulations is still present. One alternative to cationic lipids is the use of formulations based on cationic polymers and non-ionic surfactant agents to fine-tune the net charge. Cationic polymers are able to interact with DNA and RNA molecules with high efficiency and thus generate complexes with a relatively small size. This particularity makes cationic polymers useful non-viral carriers for improving gene transfection efficiency [12,13]. Although poly(ethylenimine) (PEI) and poly(*L*-lysine) (PLL) are the most well-known and used polymers for gene therapy, the synthesis and efficiency of novel cationic polymers to interact with nucleic acids in order to mediate cellular uptake have been recently reviewed [13,14].

**Figure 1:**
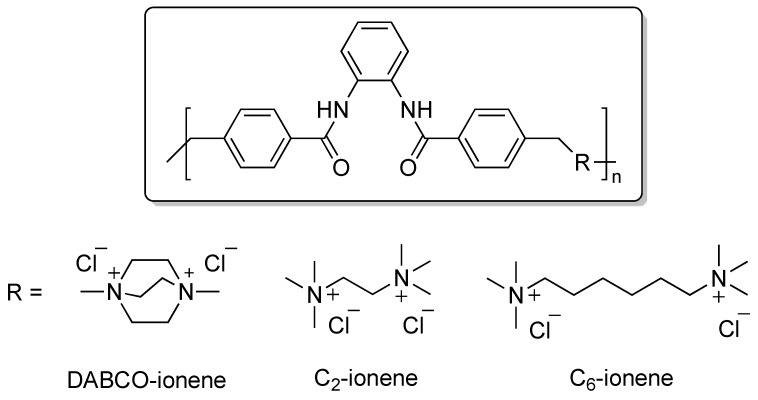
Structures of ionene polymers used in this study with charged diammonium moieties with different structures and chain length.

Ionenes are synthetic polycations with quaternary ammonium functions, which are distributed along the backbone [15,16]. In general, they can be synthesized by (a) self-polyaddition of aminoalkyl halides, (b) Menshutkin reaction between bis-tertiary amines and activated dihalide compounds or (c) via cationic functionalization of precursor polymers [17,18]. Inspired by our results which described the self-assembly properties of 1,4-diazabicyclo[2.2.2]octane (DABCO) derived ionene [19] as well as dye uptake [20] and phase-transfer catalysis [21], we decided to take advantage of the positively charged diammonium moieties present in ionene structures as cationic monomers to favor the electrostatic interaction process with nucleic acids [22,23,24,25]. In this work we have prepared surfactant-ionene formulations with a series of ionenes made of *N*,*N′*-*(o*-phenylene)dibenzamide (**1**) and α,ω-tertiary diamines (Figure 1) with the aim to further study the DNA complexation in terms of zeta potential, small-angle X-ray scattering (SAXS) and effect of transfection efficiency potency in antisense technology [1] by studying the ability of the polyplexes depending on the linker length and flexibility of the polymers to inhibit the *Renilla* luciferase gene. As mentioned above, the use of a non-ionic surfactant in the formulation (Tween 80) aims to avoid interactions with plasma proteins [12] and cell toxicity observed for cationic lipids. Our findings showed that the cationic ionene polymers formulated with a non-ionic surfactant were not toxic and were able to deliver antisense oligonucleotides into cells, thereby inhibiting the luciferase expression with promising efficiencies. These results open new insights on the design of novel ionene-based non-viral carriers for nucleic acids.

## 2. Results and Discussion

### 2.1. Size and ζ-Potential Measurements

Cationic vesicles containing the respective ionene polymer and polysorbate 80 as a non-ionic surfactant agent were prepared by the thin-film hydration method in 20 mM of 4-(2-hydroxyethyl)-1-piperazineethanesulfonic acid; HEPES (pH 7.4) buffer solution. A fixed amount of an antisense oligonucleotide (0.5 µM, *Luc*) was used to obtain a series of polyplexes at several N/P ratios (ratio of nitrogen of polymer to phosphate of DNA) that ranged from 0.05 to 6. Finally, the surface charge was measured in order to determine the overall charge of the preformed ionene polyplexes in solution.

**Figure 2:**
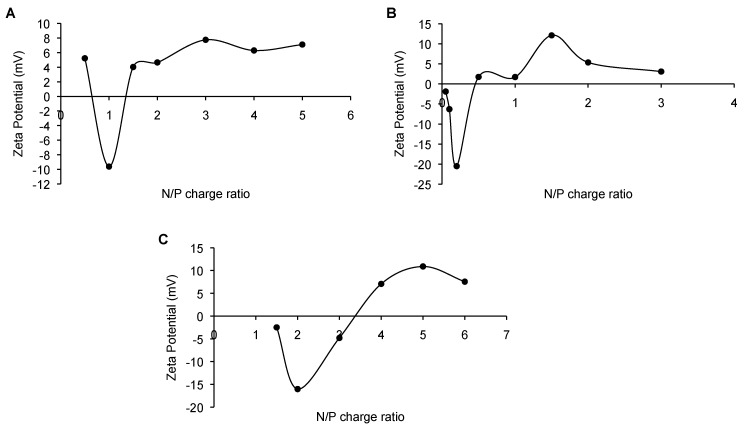
ζ potential of polyplexes at several N/P charge ratios derived from ionene polymers, antisense oligonucleotide and polysorbate 80. (**A**) DABCO-ionene; (**B**) C_2_-ionene; (**C**) C_6_-ionene.

As depicted in Figure 2, a transition from negative to positive values was clearly observed with an increase of the ionene concentration of the prepared cationic vesicle formulations. This change suggested the evidence of electrostatic interactions among negatively charged oligonucleotides and positively charged vesicles, which led to the formation of the expected ionene-based polyplexes. Interestingly, we noticed that this transition was different depending on the formulation used. Thus, in case of DABCO-ionene the formulations reached a positive net charge at N/P ratios ≥1.5; whereas for C_2_- and C_6_-ionene was around ≥0.5, 1 and 4, respectively. All formulations reached a plateau when the surface charge density was compensated by an increase in the number of cationic polymer chains in the three systems (N/P ratio of 2, 4 and 6).

Surprisingly, we observed similar trends in the behavior of the systems when ionene formulations were added at lower N/P ratios. In particular, an initial decrease in the charge of the complexes was detected upon the addition of the cationic polymers, followed by a charge increase as described before. This initial decrease in the charge ratio was also observed and described with other families of cationic polymers, such as DNA-PEI complexes [26]. Similarly, the presence of initial amounts of cationic polymers in the three systems may result in varying the degree of the folding of DNA molecules by decreasing the amount of free DNA, therefore leading to an increase in the concentration of the negative charge. This effect was primarily observed when the first ionene-based polymers were added to these three systems.

### 2.2. SAXS Measurements

To further investigate the structural information of our preformed ionene polyplexes, solutions of DNA from salmon testes in 20 mM of HEPES (pH 7.4) were put in contact with two representative ionene-based vesicle formulations (C_2_- and C_6_-ionene). The expected viscous complexes were formed, separated from the excess solution and washed with deionized water. The first attempts to visualize the scattered X-ray intensities were unsuccessful because the scattering signal was too weak for both complexes (data not shown). It is well known that DNA can be compacted by cationic surfactants forming lipoplexes, which can then co-precipitate at high concentrations to form gels with strong mechanical properties. When a drop of a concentrated DNA solution is put in contact with a concentrated cationic surfactant solution, macroscopic films can be obtained that surround the droplet. Interestingly, these films are quite consistent as they can be cut and opened. Furthermore, the films can be also dried and rehydrated, preserving their main structural properties as observed by SAXS [27]. Thus, to get better resolution and enhance the signal spectra, the resultant two complexes were further concentrated by evaporation. This process significantly showed the presence of one peak, which could be assigned to the pre-formed DNA complexes with C_2_- and C_6_-ionene, respectively.

**Figure 3:**
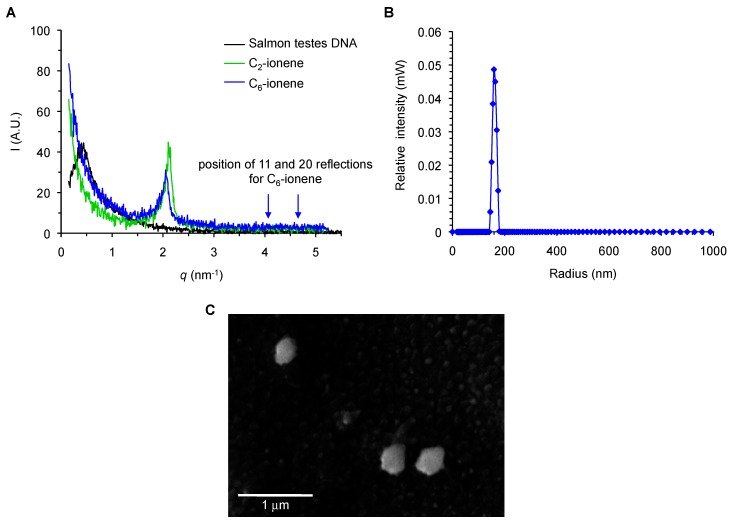
(**A**) Scattered X-ray intensity as a function of scattering vector *q* at 25 °C. The complex for C_2_-ionene (green) and C_6_-ionene (blue) are shown together. The scattering pattern produced by the salmon testes DNA (black) is also included as a control. The expected positions of second and third reflexions for hexagonal packing are shown for C_6_-ionene as arrows; (**B**) Representative dynamic light scattering DLS measurement of polyplex made of C_6_:*Luc* oligonucleotide; (**C**) Representative cryo-scanning electron microscopy image of polyplexes based on C_6_-ionene.

As illustrated in Figure 3A, scattered X-ray intensities of the two ionene complexes showed the presence of single peaks in which their position did not differ significantly among each other (*q* = 2.402 and 2.306 nm^−1^ for polyplexes based on C_2_- and C_6_-ionene, respectively). Disappointingly, we were not able to detect the presence of a second and third peak throughout the spectra, which might confirm a hexagonal packing of our pre-formed complexes. Additionally, the scattering pattern for the DNA from salmon testes (without forming complexes) was also included in the spectra for reference as a control. However, if we assume hexagonal symmetry as observed in lipoplexes [28], the distance calculated between the centers of the cylinders is 3.0 nm for C_2_-ionene and 3.1 nm for C_6_-ionene. By comparing these results with the DNA complexes made using other kinds of surfactant agents, we see that both ionene complexes are much more compact. For example, the observed distances between cylinders were between 4.2 and 4.7 nm in the case of several lipo-amino acid derivatives whereas the distance for cetyl trimethylamonium bromide (CTAB) and myristyltrimethyl ammonium bromide (MTAB) were 6.5 and 5.68 nm, respectively [29]). If we tentatively compare these distances with the dimensions of a DNA double helix (diameter between 2.2 and 2.6 nm [30]), it seems that the structure of ionene-based complex might pack in a different way. Taking into consideration that DNA chains can form hexagonal structures intertwined with cationic lipid cylindrical micelles, the observed distances in the case of ionene complexes suggest diameters of 1.2 and 1.4 nm for C_2_- and C_6_-ionene cylinders, respectively, with a 2:1 hexagonal. These diameters seem reasonable for these molecules.

Finding that SAXS measurements showed polyplex structures formed by the formulation based on C_2_- and C_6_-ionene and DNA salmon testes were similar, the polyplex made of the C_6_:*Luc* oligonucleotide was chosen as a representative compound to carry out the corresponding dynamic light scattering (DLS) measurements. As illustrated in Figure 3B, polyplexes were formed and showed an average diameter size of 320 ± 5 nm with good degree of dispersity (0.3). Additionally, polyplexes based on C_6_-ionene were characterized by a cryo-scanning electron microscopy (cryo-SEM) (Figure 3C). The cryo-SEM analysis revealed spherical morphologies of the particles with similar average sizes (340 nm) than those achieved through the use of DLS, as previously described. These results show the appropriateness of the use of C_6_-ionene polymer as a non-viral vehicle for nucleic acids (e.g., phosphorothioate and siRNA oligonucleotides).

### 2.3. Cytotoxicity Assay

Prior to transfection experiments, the cytotoxicity of polyplexes (containing the antisense oligonucleotide, *Luc*) derived from the different ionenes was tested on the HeLa cells. Three concentrations were evaluated (60, 120 and 300 nM) with respect to the *Luc* oligonucleotide at N/P ratios of 2, 4 and 6, respectively. As a control, the effect on the HeLa cells viability of the three cationic vesicle formulations (DABCO-ionene, C_2_-ionene and C_6_-ionene; *mock*) without forming complexes with *Luc*, was also tested at 0.6, 1.20 and 3.0 µM (concentrations that were used for the preparation of the polyplexes). Cells were incubated with the respective formulations at 37 °C for 24 h and normalized viabilities of treated cells with regard to untreated cells were measured by using a tetrazolium-based colorimetric (MTT) assay [31].

**Figure 4:**
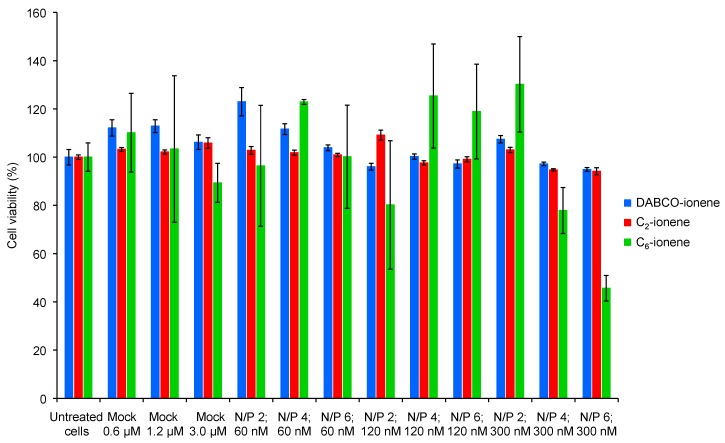
Normalized cell viability of polyplexes derived from ionenes, antisense oligonucleotide and polysorbate 80. Toxicity of cationic vesicles without forming complexes was also tested. Polyplexes were tested at 60, 120 and 300 nM (concentration of *Luc* oligonucleotide) with N/P ratios of 2, 4 and 6. Ionene-based polymers (DABCO, C_2_ and C_6_, respectively) without forming polyplexes were tested at 0.6, 1.2 and 300 µM, respectively). DABCO-ionene = blue color, C_2_-ionene = red color, C_6_-ionene = green color. Each value represents the mean of at least 5 measurements.

As displayed in Figure 4, vesicle formulations that did not contain the *Luc* oligonucleotide showed no cytotoxicity at the three concentrations tested (cellular viabilities >90%). Also, polyplexes containing 60 nM of *Luc* proved to be harmless to the HeLa cells at N/P ratios of 2, 4 and 6, respectively. With respect to *Luc*, the formulations derived from DABCO- and C_2_-ionene still showed perfect nontoxic behavior at concentrations of 300 nM. However, those derived from C_6_-ionene based formulations showed a clear decrease in cell viability with rising N/P ratios (from N/P = 4: 78% to N/P = 6: 46%). Additionally, we also evaluated the toxicity caused by the preformed lipoplexes based on lipofectamine and the *Luc* oligonucleotide as controls. As displayed in Figure S1 (Supporting Information), polyplexes obtained at 60 nM of the *Luc* oligonucleotide showed low toxicity levels whereas the presence of polyplexes obtained at 120 and 300 nM of the *Luc* oligonucleotide was detrimental to cellular viability (55% in both cases). This result confirmed the data obtained by microscopy in which changes in the morphology of the cells were observed after treatment with lipoplexes at 300 nM (Figure S2).

### 2.4. Transfection

**Figure 5:**
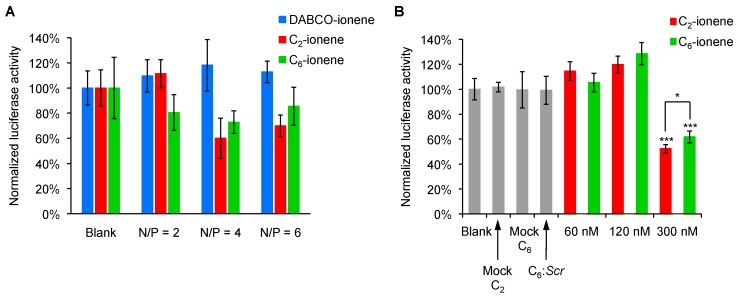
Normalized gene-specific silencing activities targeting *Renilla* luciferase mRNA for polyplex formulations. (**A**) In the absence of fetal bovine serum (FBS), at different N/P ratios at 120 nM and (**B**) in the presence of FBS at concentrations of 60, 120 and 300 nM. Data are expressed as mean values of three replicates (±SD); * and *** indicate *p* < 0.05 and *p* < 0.001, respectively.

The described ionene-based formulations (DABCO-, C_2_- and C_6_-ionene) containing an antisense oligonucleotide (*Luc*), a 17-mer complementary to the mRNA *Renilla* luciferase gene, have been tested regarding their ability of silencing luciferase activity (Figure 5). Generally, cationic particles are prone to interact with negatively charged proteins which may initiate aggregation processes leading to increasing particle sizes that may abolish the effectiveness of gene transfection [32]. To avoid this undesirable effect, initial transfection experiments mediated by ionene formulations were evaluated in the absence of serum proteins (Figure 5A). Thus, cationic polyplexes at concentrations of 120 nM with respect to *Luc* oligonucleotide at N/P ratios of 2 and 4 (N/P ratio of 6 was dismissed according to MTT data) were prepared.

Interestingly, two factors influenced the transfection process: the ionene structure and the relationship between ionene molar and charge ratio. According to Figure 5A, the use of the DABCO ionene-based formulation was detrimental in the transfection at the two N/P ratios tested, possibly due to the rigid linker which might have a detrimental effect on complex formation with oligonucleotides and therefore a negative effect on mediating nucleic acid delivery. However, moderate gene silencing activities were obtained with cationic formulations at a N/P ratio of 4, under serum-free conditions at 120 nM with knockdown values of 40% and 27% for C_2_- and C_6_-ionene, respectively which inhibited luciferase production. This silencing activity, though modest, might come from the release and discharge of the *Luc* oligonucleotide from endosomes, most likely by protonation mechanisms [33].

As C_2_- and C_6_-ionene formulations at a N/P ratio of 4 showed the best transfection profile and lowest toxicity at 120 nM, they were used for investigating the gene-silencing abilities in the presence of 10% fetal bovine serum (FBS) (Figure 5B) and at concentrations of 60, 120 and 300 nM with respect to the *Luc* oligonucleotide. According to the results, transfection efficiencies strongly depended upon the presence of the serum, which tended to interact eagerly with our two cationic polyplexes. This interaction resulted in decreasing the transfection efficiency of the formulations. Thus, while C_2_- and C_6_-ionene polyplexes silenced the *Renilla* luciferase 40 (and 27% at 120 nM in the absence of FBS), the presence of FBS was detrimental on inhibiting luciferase at the same concentration. Curiously, the values obtained for the two ionenes (C_2_ and C_6_) at 120 nM exceeded approximately 10–20% of the values obtained for untreated cells (*blank*) according to the dual-luciferase reporter assay. It is undeniable that these results confirm our ionene-based polyplexes were not able to impart cellular uptake and therefore inhibit luciferase production at 120 nM. However, we are unable to demonstrate whether there might have been undesirable interactions among the ionene-based polymers and the *Renilla*/Firefly luciferases at the aforementioned concentration. However, it is also noteworthy that this anomalous behavior was occasionally observed with other non-viral carriers, such as the proline-rich cell-penetrating peptides [34] and carbosilane dendrons [35], as vehicles to transport antisense oligonucleotides. 

When the concentration of the *Luc* oligonucleotide was increased up to 300 nM, both ionene formulations (C_2_- and C_6_-ionene) were able to promote gene delivery with similar and significant efficiencies (48% and 38% luciferase inhibition for C_2_- and C_6_-ionene, respectively) (*** *p* < 0.001). Interestingly, these silencing activities, though similar, were also found to be statistically significant in the case of C_2_-ionene formulations when compared to C_6_-ionene formulations (* *p* < 0.05). It seems that a medium-sized chain length of the linker has a beneficial effect on the transfection ability; perhaps the proper balance between rigidity and flexibility of the polymers must be found for optimal effects. Additionally, a scramble (*Scr*) oligonucleotide complexed with C_6_-ionene formulation at the same N/P ratio of 4 together with C_2_- and C_6_-ionene formulations without forming polyplexes (mock) were used as controls and did not produce any effect on luciferase inhibition, as expected. Finally, to determine the specificity of the gene transfection process, the *Luc* and *Scr* oligonucleotides (60 nM) were formulated into liposomes as controls (in the presence of lipofectamine), obtaining similar inhibition results as previously reported [34] (Supporting Information; Figure S3).

## 3. Materials and Methods

### 3.1. Materials

Polysorbate 80 (Tween 80), 3-(4,5-dimethylthiazol-2-yl)-2,5-diphenyltetrazolium bromide (MTT reagent), sodium salt of DNA from salmon testes (polymerization average degree of 2000 base pairs) and antisense oligonucleotide of sequence (5′-CGT TTC CTT TGT TCT GGA-3’; *Luc*) were purchased from Sigma-Aldrich (St. Louis, MO, United States) and used as received. A scramble sequence (5′-CTG TCT GAC GTT CTT TGT-3′; *Scr*) was synthesized in-house and purified by dimethoxyltrityl, DMT off-based protocols. Lipofectamine 2000 was purchased from Invitrogen (Carlsbad, CA, USA). Phosphate-buffered saline (PBS) buffer and Dulbecco’s Modified Eagle’s Medium (DMEM), which was supplemented with a 10% heat-inactive fetal bovine serum (FBS) along with distilled water (DNAse/RNAse free) were purchased from Gibco (Waltham, MA, USA). Additional nuclease-free water was also prepared by using 0.1% of diethylpyrocarbonate (DEPC) to ensure the removal of RNase contamination, as well as autoclaved and filtered before using. Luciferase assay kits were purchased from Promega (Madison, WI, United States). Qiagen Giga plasmid purification kit was purchased from Qiagen (Hilden, Germany). Starting materials for polymer synthesis were purchased from Sigma Aldrich (St. Louis, MO, USA) or TCI (Zwijndrecht, Belgium). Luminiscence values were measured in a Promega Glomax Multidetection instrument (Madison, WI, USA). All ionene polymers were synthesized by step-growth polymerization following the two-step procedure previously described and showed the same spectroscopic data [19,20,21,36]. In short, the reaction of *o*-phenylenediamine with 4-(chloromethyl)benzoyl chloride in the presence of Et_3_N in CH_2_Cl_2_ afforded the corresponding bis-benzamides in good yields (75–98%). Subsequent copolymerization with equimolar amounts of the desired α,ω-diamine in dimethylformamide (DMF) at 80 °C provided white precipitates within 2–6 days. The precipitates were filtered, washed (with DMF, CH_3_CN and CH_2_Cl_2_) and dried under vacuum to afford the ionenes in modest yields (43–80%). Sufficient solubility for gel permeation chromatography (GPC) was achieved by exchanging the chloride anions by bis(trifluoromethanesulfonyl)amide (TFSA) anions using lithium bis(trifluoromethanesulfonyl)azanide (LiTFSA) in hot water. These ionenes·TFSA showed low degrees of polymerization (*n* = 7–14) and high dispersity values (*Đ* = 2.1–3.1). Weight-average molecular weights, *M*_w_ (Da), for ionenes·TFSA: 8.1 × 10^3^ (DABCO-ionene); 1.6 × 10^4^ (C_2_-ionene); 6.9 × 10^4^ (C_6_-ionene); number-average molecular weight, *M*_n_ (Da), for ionenes·TFSA: 3.9 × 10^3^ (DABCO-ionene); 6.1 × 10^3^ (C_2_-ionene); 2.2 × 10^3^ (C_6_-ionene).

### 3.2. Preparation of Polyplexes

Cationic vesicles were prepared by mixing ionenes (1–2 mg) and polysorbate 80 in a 1:1 (*n*/*n*) molar ratio and dissolving/suspending those in MilliQ water (1 mL). Mixtures were dried in a speed vacuum at 45 °C for 18 h. The resulting film was rehydrated in 1 mL of sterile HEPES buffer (20 mM; pH 7.4), incubated at 60 °C for 20 min, sonicated for 3 min and filtered through a 13 mm nylon filter with 0.2 µm pore width. Polyplexes were obtained by adding an oligonucleotide stock solution to the cationic vesicle solutions to give N/P ratios ([cationic amino groups]_ionene_/[anionic phosphate groups]_nucleic acid_) between 0.05 and 8. The mixtures were vortexed, sonicated for 10 s and finally incubated at 37 °C for 30 min.

The synthesized ionene monomers each contained two terminal amine groups that were positively charged at physiological pH conditions (7.4). The antisense oligonucleotide (*Luc* oligonucleotide) contains 17 negative charges and the N/P ratio can be calculated by using Equation (1), as described elsewhere [37].

(1) $$N/P\;\mathrm{ratio}=\frac{\mathrm{number}\;\mathrm{of}\;\mathrm{moles}\;\mathrm{of}\;\mathrm{ionene}\;\mathrm{monomer}\times 2}{\mathrm{number}\;\mathrm{of}\;\mathrm{moles}\;\mathrm{of}\;Luc\;\mathrm{oligonucleotide}\times 17}$$

### 3.3. Zeta Potential

The zeta potential values were obtained by laser Doppler velocimetry by using a Zetasizer Nano ZS (Malvern Instruments; Malvern, UK) equipped with a He–Ne red light laser (*λ* = 633 nm). Polyplex solutions were measured at an oligonucleotide concentration of 0.5 µM and N/P ratios from 0.05 to 6 at 25 °C. Samples (50 µL) were diluted in 0.1 mM NaCl solution (950 µL) and introduced into folded capillary cells. The Smoluchowski approximation was used to calculate the ζ potential values. Measurements were run in triplicate.

### 3.4. Size Measurements

The hydrodynamic diameter of polyplexes (cationic ionene/DNA complexes) was determined by a dynamic light scattering (DLS) spectrometer (LS Instruments, 3D-cross correlation multiple scattering) equipped with a He–Ne laser (632.8 nm). Measurements were carried out at a scattering angle of 90°, in triplicates at 25 °C without sonication. The particle radii were calculated by fitting the first cumulant parameter.

### 3.5. Small-Angle X-ray Scattering (SAXS) Measurements of Ionene Polyplexes

Small-angle X-ray scattering measurements were recorded on S3-MICRO (Hecus X-ray system, GMBH, Graz, Austria) which was coupled to a GENIX-Fox 3D X-ray source (Xenox, Grenoble) and coupled with a 3D-focusing mirror, which produced a detector-focused X-ray beam with *λ* = 0.1542 nm Cu *K*_α_-line (greater than 97% purity and less than 0.3% of *K*_α_). Transmitted scattering was measured by using a PSD 50 Hecus in the SAXS regime (0.09 nm^−1^ < *q* < 6 nm^−1^) including Bragg peaks and diffuse scattering. Measurements were carried out at a constant temperature (25 °C) for 24 h. Detector counting was accumulated for 20 min intervals; if no temporal variation was observed, several runs were summed up to reduce the noise of background level. A stock concentration of DNA from salmon testes in 20 mM of HEPES (pH 7.4) was properly mixed with a stock concentration of a surfactant-ionene formulation in 20 mM of HEPES buffer at 37 °C for 30 min. The resultant polyplexes (DNA: ionene complexes) were isolated, dried and deposited in a glass capillary of 1 mm diameter with 10 µm wall thickness. SAXS scattering curves were plotted as a function of the scattering vector modulus (Equation (2)) where θ is the scattering angle and *λ* the incident radiation wavelength. A silver behenate sample was used in order to calibrate the system scattering vector.

(2) $$q=\frac{4\pi }{\lambda }\sin\frac{\theta }{2}$$

### 3.6. Cryo-Scanning Electron Microscopy (cryo-SEM)

Polyplexes made of an ionene-C_6_ based formulation and the *Luc* oligonucleotide were obtained at a N/P ratio of 4. The resultant polyplexes were rapidly frozen in liquid nitrogen and cut with an equipped cold knife. Polyplexes were sputered with gold palladium, observed and processed by using a Hitachi S-3500N (Hitachi High-Technologies Corp., Tokyo, Japan) scanning electron microscope operated at 5 kV.

### 3.7. Cytotoxicity Assay

The adherent cell line HeLa (human carcinoma) was plated at a density of 5 × 10^3^ cells/well (50% confluency) on a 96-well plate in 200 µL of Dulbecco’s Modified Eagle’s medium (DMEM), supplemented with 10% fetal bovine serum (FBS), in the absence of antibiotics. The HeLa cells were incubated at 37 °C in a humidified atmosphere (5% CO_2_) for 24 h to permit cell attachment. Then the medium was removed and replaced by fresh one to give a final concentration of 200 µL. Polyplexes and cationic vesicles were prepared as described above and added to the wells to give concentrations of 60, 120 and 300 nM and N/P ratios of 2, 4 and 6. The mixture was incubated at 37 °C for 18 h, then the medium was removed and the wells were washed with PBS (1 × 200 µL). Fresh DMEM was added (200 µL) and cells were incubated again at 37 °C for 3 h. MTT solution (10 µL; 5 g/L in PBS buffer) was added to each well and plates were stored at 37 °C for further 2.5 h. Then medium was removed, DMSO (100 µL) was added and absorbance was measured at 570 nm. Each measurement was performed with six replicates. Additionally, to evaluate the effect of lipofectamine in the HeLa cells, the *Luc* oligonucleotide was mixed at 60, 120 and 300 nM, respectively at room temperature with lipofectamine, according to the manufacture’s protocol. HeLa cells and the corresponding lipoplexes were incubated following the same experimental procedure as described before.

### 3.8. Gene Transfection and Antisense Technology Studies

#### 3.8.1. In the Absence of FBS

The HeLa cells were regularly passaged to maintain exponential growth. Cells were seeded the day before at a density of 10^5^ cells/well on a 24-well plate, with each well in DMEM (supplemented with 10% FBS). Cells were incubated at 37 °C for 18 h in a humidified atmosphere (5% CO_2_) to permit cell attachment. The medium was then replaced by fresh one (500 µL). A solution containing firefly luciferase (pGL4) (30 µL; 100 ng/µL) and *Renilla* luciferase (30 µL; 10 ng/µL) in Opti-MEM™ (90 µL) was combined to a solution containing Lipofectamine 2000 (3.9 µL; 1 mg/mL) in Opti-MEM™ (146.4 µL). The resultant formulation was incubated for 10 min at room temperature. One hundred µL of this formulation was added to each well and the cells were incubated at 37 °C for 6 h. The medium was removed, the cells were washed with PBS (2 × 400 µL) and DMEM without FBS was added (500 µL). Polyplexes were added (100 µL) to give final oligonucleotide concentrations of 120 nM and N/P ratios of 2, 4 and 6 and cells were incubated at 37 °C for 24 h. The medium was removed, the cells were washed with PBS (1 × 500 µL) and the cells were stored at −20 °C for 18 h. After thawing the cells, 100 µL of lysis buffer was added to each well. The plate was shaken at 480 rpm at room temperature for 15 min. Lysates (25 µL) were transferred to a 96-well plate and luminescence was recorded after adding, alternatingly, Luciferase assay reagent (25 µL) and Stop & Glow reagent (25 µL). Inhibition results generated by polyplex-based ionenes were expressed as normalized ratios between the reported luciferase (*Renilla*) and control luciferase (Firefly).

#### 3.8.2. In the Presence of FBS

The HeLa cells were regularly passaged to maintain exponential growth. Cells were seeded the day before at a density of 10^5^ cells/well on a 24-well plate each well in DMEM supplemented with 10% FBS. The cells were incubated at 37 °C for 18 h. The *Renilla* and Firefly luciferases were transfected following the same procedure as described above. After 6 h of incubation at 37 °C, the medium was removed, cells were washed with PBS (2 × 400 µL), and DMEM supplemented with 10% FBS was added (500 µL). Polyplexes were added to give a final N/P ratio of 4 and oligonucleotide concentrations of 60, 120 and 300 nM. The cells were incubated at 37 °C for 24 h. The medium was removed, cells were washed with PBS (1 × 500 µL) and stored at −20 °C for 18 h. After thawing the cells, 100 µL of lysis buffer were added to each well. Luminiscence was measured following the same protocol as described before. Inhibition results generated by polyplex-based ionenes were expressed as normalized ratios between the reported luciferase (*Renilla*) and control luciferase (Firefly). *Luc* and *Scr* oligonucleotides at 60 nM were formulated into liposomes (in the presence of lipofectamine) as positive and negative controls, respectively.

Statistical Analysis. Data were shown as mean ± standard deviation (SD) and are the result of an average of three replicates. Statistical differences were determined by using Student’s *t*-test and were considered significant when * *p* < 0.05 and *** *p* < 0.001.

## 4. Conclusions

Cationic vesicles derived from ionene polymers with an alternating α,ω-tertiary diamine linker and the non-ionic surfactant polysorbate 80 were used to entrap oligonucleotide single strands which were complementary to *Renilla* luciferase mRNA. The zeta potential of the vesicle formulations at different N/P ratios ([cationic amino groups]_ionene_/[anionic phosphate groups]_nucleic acid_) was measured to ensure a positive charge of the system. The MTT assay showed no toxicity for formulations derived from DABCO- and C_2_-ionene at several concentrations and N/P ratios (2, 4 and 6) whereas C_6_-ionene showed critical cell viabilities at 300 nM with a N/P ratio of 6 (46% of cell viability). Transfection experiments of all cationic vesicle formulation were investigated by performing a luciferase activity assay at 120 nM and N/P ratios of 2 and 4 in the absence of proteins. As a result, DABCO-ionene derived formulations showed no transfection ability at both N/P ratios tested. The contrast for C_2_- and C_6_-ionene derived formulations showed perfect non-toxic behavior, and the luciferase activity was reduced by 48 and 38% respectively, at a N/P ratio of 4 and *Luc* concentration of 300 nM. The use of this complete, harmless formulation at higher concentrations might be an interesting alternative to current transfection agents. Further structure–activity relationships need to be carried out in order to obtain optimized cationic ionene derivatives that are less susceptible to FBS proteins. In addition, further studies to determine the effect of the polymer topology (positional isomers) on the transfection efficiency are under way in our laboratories and the results will be reported in due course.

## Supplementary Materials

Supplementary materials can be found at http://www.mdpi.com/1422-0067/18/6/1139/s1.
