# Supplementary material for: Transfection of Antisense Oligonucleotides Mediated by Cationic Vesicles Based on Non-Ionic Surfactant and Polycations Bearing Quaternary Ammonium Moieties
Source: Int J Mol Sci. 2017 May 26;18(6):1139. doi: 10.3390/ijms18061139 (PMC5485963; doi:10.3390/ijms18061139)
Supplement: Supplementary file 1 [file ijms-18-01139-s001.pdf]

Transfection of Antisense Oligonucleotides Mediated by Cationic Vesicles Based on Non-Ionic Surfactant and Polycations Bearing Quaternary Ammonium Moieties

Judith Mayr, Santiago Grijalvo, Jürgen Bachl, Ramon Pons, Ramon Eritja and David Díaz Díaz

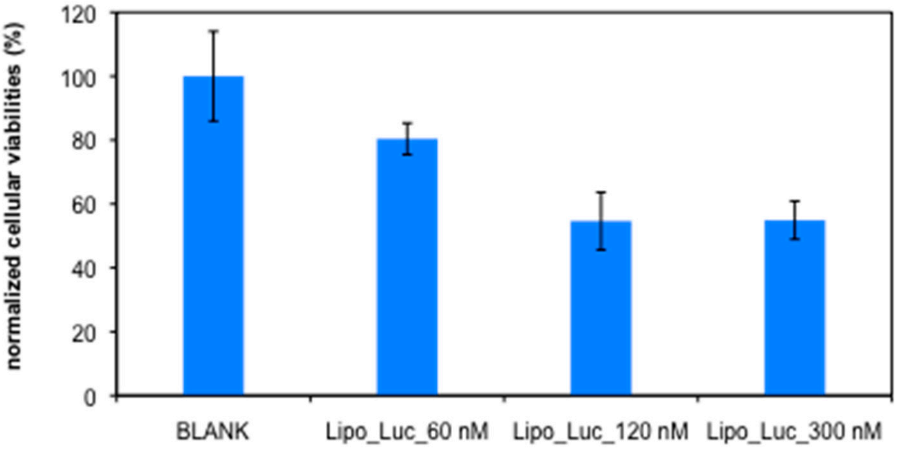

Figure S1. Cellular viabilities of preformed lipoplexes made of lipofectamine and *Luc* oligonucleotide at three concentrations (60, 120 and 300 nM, respectively). Each value represents the mean of 6 measurements.

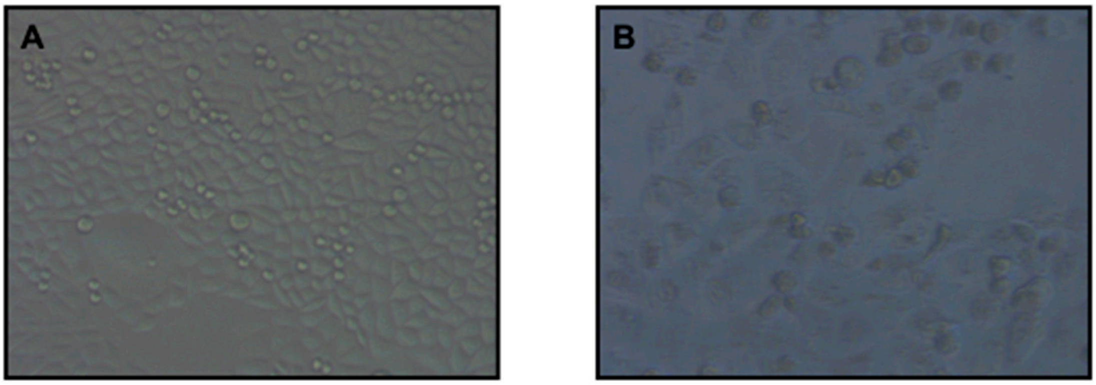

Figure S2. HeLa cell images of untreated cells (**A**) and cells treated with lipoplexes made of lipofectamine and *Luc* oligonucleotide at 300 nM after 18 h incubation (**B**).

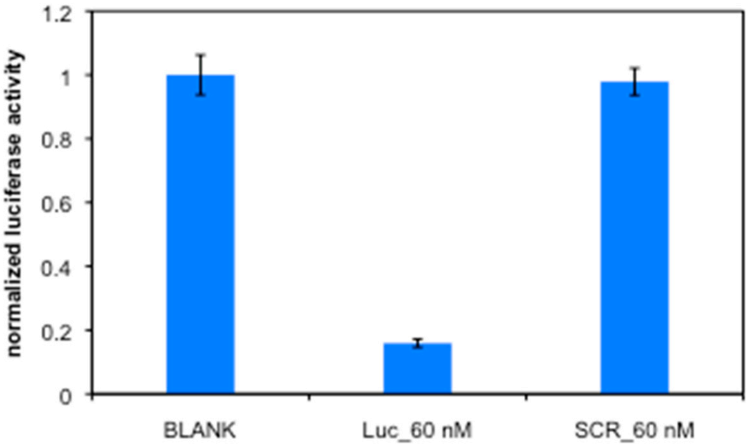

Figure S3. Plot of gene-specific antisense activity for *Luc* oligonucleotide at 60 nM in the presence of lipofectamine. Gene-knockdown specificity was confirmed with a scrambled sequence (*Scr*) at 60 nM in the presence of lipofectamine. Transfection experiment was carried out in the presence of formulated liposomes made of *Luc* and the corresponding plasmids *Renilla* and Firefly luciferases. Bars indicate mean ± S.D. (n = 3 independent treatments).
